# Prevalence and associated factors of depression, anxiety, and stress among high school students in, Northwest Ethiopia, 2021

**DOI:** 10.1186/s12888-022-04393-1

**Published:** 2022-11-28

**Authors:** Girum Nakie, Tesfaye Segon, Mamaru Melkam, Getachew Tesfaw Desalegn, Tadele Amare Zeleke

**Affiliations:** 1 Department of Psychiatry, College of Medicine and Health Science, University of Gondar, Gondar, Ethiopia; 2 Departments of Psychiatry, College of Medicine and Health Science, Metu University, Metu, Ethiopia

**Keywords:** Depression, Anxiety, Stress, High school, Students, Ethiopia

## Abstract

**Background:**

Many studies have revealed that students’ performance in school, is affected by symptoms of depression, anxiety, and stress, which may impair their academic achievement, and lead to school dropout. However, to date, no studies have evaluated these three disorders among high school students in Africa. Therefore, in this study, we aimed to assess the prevalence of depression, anxiety, stress, and their associated factors among high school students in Northwest Ethiopia.

**Methods:**

An institution-based cross-sectional study was conducted. A simple random sampling technique was used to select 849 participants from six high schools in Northwest Ethiopia. A self-administered Depression, Anxiety, and Stress Scale (DASS-21) questionnaire was used to collect the data. Data were analyzed using SPSS Version 25.0 software to identify factors associated with DAS, and bi-variable and multi-variable analyses were performed.

**Results:**

The prevalence of depression, anxiety, and stress was 41.4, 66.7, and 52.2% respectively. Being female (AOR = 1.304, 95% CI = 1.006–1.849), higher risky khat chewers (AOR = 5.595, 95% CI = 2.357–11.132), having social phobia (AOR = 1.416, 95% CI = 1.045–1.919) were associated with depression. Being higher risky cigarette smokers (AOR = 4.777, 95% CI = 1.407–7304), having a history of chronic medical illness (AOR = 2.099, 95% CI = 1.045–4.218), and having a family history of mental illness (AOR = 1.777, 95% CI = 1.028–3.073) associated with anxiety**.** Stress was associated with high-risk alcohol drinkers (AOR = 1.828, 95% CI = 1.012–3.303), rural residency (AOR = 1.395, 95%CI = 1.010–1.925), and low social support (AOR 1.7391, 95% CI = 1.203–2.515).

**Conclusion:**

The burden of DAS among high school students was found to be high. Female sex, chewing khat, and having social phobia are associated with depression. Conversely, smoking cigarettes, having a chronic medical illness, and having a family history of mental illness are all linked to anxiety. Being a highly risky alcoholic drinker, having poor social support, and being a rural resident are positively associated with stress. Therefore, extending mental health services to all high schools, and strengthening the existing counseling services, are recommended.

## Introduction

Students adapt to various psychosocial changes besides coping with the academic and social demands of their future professional careers [1]. The high expectations of academic achievement have created a very stressful environment that if left untreated, can be hazardous to their physical and mental health [2, 3]. The reported prevalence of depression, anxiety, and stress y between different studies and from place to place [4, 5]. A survey conducted in 17 countries of adolescents found that, on average, 1 in 20 people reported having an episode of depression. It is the leading cause of disability and the fourth leading contributor to the global burden of disease [6]. According to the Royal Society for Public Health and the Young Health Movement, the problem has increased by 70% in the past 25 years in young people [7]. In developing countries, the burden of depression, anxiety, and stress is also growing among adolescents [4, 8, 9].

Many studies have revealed that students’ performance in school, is affected by symptoms of depression, anxiety, and stress, which may impair their academic achievement, lead to deterioration in relationships, and marital problems and affect future employment. Recently, depression is estimated to affect 350 million people worldwide [10]. It interferes with individuals’ emotional, cognitive, and social abilities, which can lead to underemployment and reduced productivity [11]. The symptoms of these three disorders can also lead to a lack of communication with friends and family members; substance abuse; feelings of abandonment; homicidal ideation; and suicidal tendency can occur [12–14].

Using the 21-item Depression, Anxiety, and Stress Scale, a cross-sectional research of 350 students at a chosen boarding school in Malaysia found that the prevalence of depression, anxiety, and stress was 39.7, 67.1, and 44.9%, respectively [15]. By using DASS 42 scale, a study conducted among 48,720 secondary school students in Baghdad, Iraq revealed that the prevalence of depression, anxiety, and stress symptoms were 29.4, 40.6, and 51.1% respectively [16]. Across sectional study was conducted to assess the prevalence of psychological health problems in Chinese adolescents during the outbreak of COVID-19 using the Patient Health Questionnaire (PHQ-9) for depressive and Generalized Anxiety Disorder (GAD-7) questionnaire for anxiety symptoms. Therefore, the prevalence of depressive, and anxiety symptoms were 43.7, and 37.4%, respectively [17]. By using the DAS scale, there were two different cross-sectional studies were conducted in India. The prevalence of depression, anxiety, and stress among 830 Manipur secondary school students was 19.5, 24.4, and 21.1%, respectively, whereas among Chandigarh students, the prevalence of DAS was 65.53, 80.85, and 47.02%, respectively [1, 18].

A cross-sectional study was conducted among 545 Saudi Arabian high school girls and revealed that the prevalence of symptoms of depression, anxiety, and stress were 41.5, 66.2, and 52.5% respectively [19]. Moreover, another study was conducted among adolescent school boys more than one-third of the participants (38.2%) had depression, while 48.9% had anxiety and 35.5% had stress [20]. A cross-sectional study was done in the al-Qassim region among 1245 secondary school students using the Patient Health Questionnaire (PHQ-9) to assess depression and the GAD7 for anxiety. The study shows that 34% were mildly depressed, 24.6% were moderately depressed, 10.4% were moderately severe depression, and 5.0% were severely depressed whereas 34.1% were having mild anxiety, 19.5%) were having moderate anxiety, and 9.8% were having severe anxiety [3].

According to an institutional-based cross-sectional study conducted among 265 undergraduate students at the Arsi University, Ethiopia, the prevalence rates of depression, anxiety, and stress were 52.3, 60.8, and 40.4%, respectively [21]. There are several factors associated with the occurrence of these disorders. Female sex [1, 15, 18], young age groups, poor parental educational states, and poor academic performance were all significantly associated with Symptoms of depression, anxiety, and stress among high school students [1, 16]. Apart from the demographic characteristics of individuals, those who have a family history of psychiatric disorder, self-harm [2, 15, 18], living with relatives or alone, having little social support, and being single were all linked to symptoms of depression, anxiety, and stress in high school students [16, 18]. Research on DAS among high school students in India and other different studies revealed that alcohol drinking, occasional cigarette smoking, and other substances are highly associated with symptoms of depression, anxiety, and stress [16, 18].

While depression, anxiety, and stress among high school students have been relatively researched in developed countries, very few studies are available in developing countries, including Ethiopia. Though these three disorders were researched on university students, according to our research engine, there was no published research in Africa among high school adolescents. Therefore, this study will assess the prevalence of depression, anxiety, and stress and various factors that might lead to early interventions for further obstacles among high school students.

## Methods and materials

### Study area and population

An institutional-based cross-sectional study was conducted in April 2021 among six high school students in Northwest Ethiopia. These are Adet, Selamber, Densa Bata, Debre Mewi, Agita, and Gosheye high schools. The study area was mainly in Amhara region, covered 1018.11 km2, and had a total population of 214,852, of whom 107,010 were men and 107,842 were women. 8.92% lived in cities, 98.19% were Orthodox Christians, 1.76% Muslims, and 99.94% belonged to the Amhara ethnic group. All high school students who attended a class during data collection time were included in the study whereas; students who were unable to communicate due to acute illness during data collection time at schools were excluded.

### Sample size determination and sampling procedure

The sample size was determined by assuming single population formula. The prevalence of depression, anxiety, and stress was taken from a previously published study in Ethiopia at Arsi University students 52.3, 60.8, and 40.4% [21]. By assuming 95%, confidence interval (CI), and margin error of 5%. Therefore for the first objective taking the prevalence of depression calculating as follows

$$\textrm{n}=\frac{\left(\textrm{Z}\alpha /2\right)\ 2\ \textrm{x}\ \textrm{p}\ \left(1-\textrm{p}\right):}{d^{2}}\kern0.22em \textrm{n}=\frac{(1.96)2\ \textrm{x}\ 0.523\ \textrm{x}.477}{\left(\ 0.05\right)2}=383$$

Hence we used multi-stage sampling with two stages, we considered the stage and multiplied the sample size by the number of stages. Therefore 383 × 2 = 766.

Including 10% of the non-response rate, the final sample size was 766 + 77 = 843. The sample size for the second and third objectives by using p the prevalence of anxiety and stress were 60.8 and 40.4%. But the calculated sample size becomes 805 and 814 respectively which are less than 843. Hence the minimum sample size for this study was 843.

A stratified multi-stage sampling technique was used. There are several schools in the region. For this study f, six governmental high schools were randomly selected in the region. Then the total sample size for the study was distributed proportionally across schools according to the number of students in each school. Within each school, the sample size was again proportionally distributed across the grades (grade nine, ten, eleven, and twelve) according to class size. Then simple random sampling technique was used to select each participant from each stratum (grade) by the computer-generated method of their identification number. Finally, the selected students from all grades were taken to one hall then the questionnaires were administered after orientation.

### Data collection tools

Data were collected using a structured self-administered questionnaire that has five parts: In part one socio-demographic characteristics such as age, sex, grade, and the like were collected by using structured questionnaires. In the second part the Depression, Anxiety, and Stress Scale (DASS-21) were used to measure depression, anxiety, and stress. This questionnaire has been validated in African countries; the subscales of depression, anxiety, and stress have Cronbach’s alpha values of 0.81, 0.89, and 0.78, respectively. Each of the three DASS-21 scales contains 7 items, divided into subscales with similar content. Participants were asked to rate their symptoms throughout the previous week in each domain, ranging from 0 (did not apply at all) to 3 (applied most of the time). Each dimension’s scores were summed. The resulting score was multiplied by two and then classified as normal, mild, moderate, severe, and extremely severe using the DASS manual. Accordingly, for participants with depression, a depression score of 0–9 was considered normal, 10–13 as mild, 14–20 as moderate, 21–27 as severe, and 28 and above as extremely severe. An anxiety score of 0–7 was regarded as normal, 8–9 as mild, 10–14 as moderate, 15–19 as severe, and 20 and above as extremely severe for persons with anxiety. A stress score of 0–14 was regarded as normal, 15–18 as mild, 19–25 as moderate, 26–33 as severe, and 34 and above as extremely severe for participants who had experienced stress [22]. Moreover, the score can also be dichotomous by considering mild to extremely severe as, having depression, anxiety, and stress and otherwise not. For instance, for the depression subscale, individuals who scored greater than or equal to 10 are considered as having depression, for the anxiety subscale individuals who scored greater than or equal to 8 are considered as having anxiety, and for the stress subscale individuals who scored greater than or equal to 15 are suggesting stress [21, 22].

Part three clinical factors like social phobia was assessed by using the social phobia Inventory (SPIN) with a score of 20 or more suggesting social phobia which is validated in Nigeria. It was used among college and high school students in Ethiopia [23, 24]. Family history of mental, suicide ideation and attempt, and having chronic medical illness were assessed by structured yes/no questions. Social support was measured by the Oslo-3 social support scale, which ranges from 3 to 14. Those respondents who scored between 3 and 8 were considered to have poor social support, a score of 9–11 was considered to have moderate social support, and a score of 12–14 was considered strong social support [25].

Substance-related factors, which comprise current use and ever use were adapted from the ASSIST (alcohol, khat, and cigarette smoking substance involvement screening Test) a well-validated instrument developed by the world health organization was used. Ever use of a substance: using at least one of any specific substances for a non-medical purpose at least once in a lifetime (alcohol, chat, tobacco, others). Current alcohol use: According to the ASSIST scale, students who scored 0–10 low risk, 11–26 moderate risk, and 27 and more highly risky drinkers in the previous three months. Students who scored 0–3 low risk, 4–26 moderate users, and 27 and more highly risky users for current cigarette and khat use [26].

### Data quality control

To control the quality of data the questionnaire was initially prepared in English, then translated into the Amharic language, and finally back to English by two language experts and psychiatrists appropriately. Three BSc nurse professionals and two BSc psychiatry profession supervisors, collected data using self-administered questionnaires. One-day training was given to those data collectors and the supervisor before the actual data collection. The questionnaire was pretested one week before the actual data collection time on 5% (*n* = 42) of the study who were not included in the main survey. Therefore, the dependent variable tool assessment (DAS) depression, anxiety, and stress Cronbach alpha were 0.72, 0.84, and 0.87 respectively. Based on the feedback obtained from the pre-test, an appropriate modification was made to the questionnaire.

### Data processing and analysis

The data was coded and entered into the computer using Epi Data version 4.2.02 and exported to the Statistical Package for Social Science (SPSS) version 25 for analysis to generate descriptive statistics: means, standard deviation, frequency, and percentages. To determine an association between dependent and independent variables adjusted odds ratios were used in Binary logistic regression and the significance level was determined using a confidence interval of 95%. Bivariate and multivariate analysis was done to identify the independent predictors of the outcome variables. Firstly, each independent variable was separately entered into the bivariable analysis. The variables with a *p*-value of less than 0.2 on bivariate analysis were entered into multivariable analysis for further analysis. Then adjusted odds ratio with 95% CI was computed for variables having a p-value less than 0.05 in multivariate analysis of the Binary logistic regression model and considered as significantly associated with the dependent variables.

## Results

### Socio-demographic characteristics of participants

Eight hundred forty-nine subjects were included in the study, and the overall response rate was 810 (96.1%). The mean age of the participants was 18.59 ± 1.792, ranging from 15 to 25 years old, and 537 (66.3%) of them were between 15 and 19 years old. More than half of 427 (52.7%) and 571 (70.5%) of the students were females and originally from rural areas, respectively. The majority of the respondents, 631 (77.9%), live with their two parents, 77 (9.5%) live with single parents, 53 (6.5%) live alone; and 49 (6.0%) of the respondents live with relatives (Table 1).

**Table 1:** Socio-demographic characteristics of participants among high school students in Northwest Ethiopia, 2021(*n* = 810)

Variables	Categories	Frequency	Percent
Sex	Male	383	47.3
	Female	427	52.7
Age	≤19	537	66.3
	≥20	273	33.7
Place of upbringing	rural	571	70.5
	Town	239	29.5
Living arrangements	Both parents (father and mother	631	77.9
	Single parents (father or mother)	77	9.5
	Alone	53	6.5
	Relatives	49	6.0
Father educational status	Unable to read and write	215	26.5
	Can read and write but not attending formal education	458	56.5
	Learned 1–8 grade	75	9.3
	Secondary education	31	3.8
	Diploma and above	31	3.8
Mother educational status	Unable to read and write	574	70.9
	Can read and write but not attending formal education	169	20.9
	Learned 1–8 grade	36	4.4
	Secondary education	16	2.0
	Diploma and above	15	1.9
Marital status	Married	42	5.2
	Single	758	93.6
	Divorced	10	1.2
Grade	9	255	31.5
	10	204	25.2
	11	196	24.2
	12	155	19.1
Academic performance	≤49.99	68	8.4
	50–74.99	504	62.2
	75–84.99	167	20.6
	≥85	71	8.8
Absenteeism	Yes	99	12.2
	No	711	87.8

### Clinical characteristics of the respondents

More than one-third of participants 302 (37.3%) had social phobia, 91 (11.2%) had a family history of mental illness, and 55 (6.3%) students had known chronic medical illness. Regarding suicidal behavior, 79 (9.8%) and 34 (4.2%) of respondents have had suicidal ideation and attempted at least once in their lifetime (Table 2).

**Table 2:** Clinical characteristics of participants among high school students in Northwest Ethiopia, 2021 (*n* = 810)

Variables	Categories	Frequency	Present
**Having known chronic medical illness**	Yes	58	7.2
	No	752	92.8
**social phobia**	Yes	302	37.3
	No	508	62.7
**Life time suicidal ideation**	Yes	79	9.8
	No	731	90.2
**Twelve month suicidal ideation**	Yes	43	5.3
	No	767	94.7
**Life time suicidal attempt**	Yes	34	4.2
	No	776	95.8
**Twelve month suicidal attempt**	Yes	18	2.2
	No	792	97.8
**Family history of mental illness**	Yes	91	11.2
	No	719	88.8
**Social support**	Poor	249	30.9
	Intermediate	310	38.3
	Strong	251	31.0

### Substance-related characteristics

Regarding substance use, 377 (46.5%) had udrunk alcohol at least once in their lifetime, whereas khat and cigarette lifetime users were 82 (10.1%) and 61 (7.5%), respectively. About three in four of the participants (77.2%) were low-risk alcoholic drinkers, whereas moderate and highly risky alcoholic drinkers were 120 (14.8%) and 67 (8.3%) respectively (Table 3).

**Table 3:** Substance related description for participants among high school students in Northwest Ethiopia, 2021 (*n* = 849)

Variables	Categories	Frequency	Percent
Alcohol	Low	625	77.2
	Moderate	121	14.9
	High risk	64	7.9
Khat	Low	748	92.3
	Moderate	24	3.0
	High risk	38	4.7
Cigarette	Low risk	757	93.5
	Moderate	20	2.5
	High risk	33	4.1

### Prevalence of depression, anxiety, and stress

**Fig. 1:**
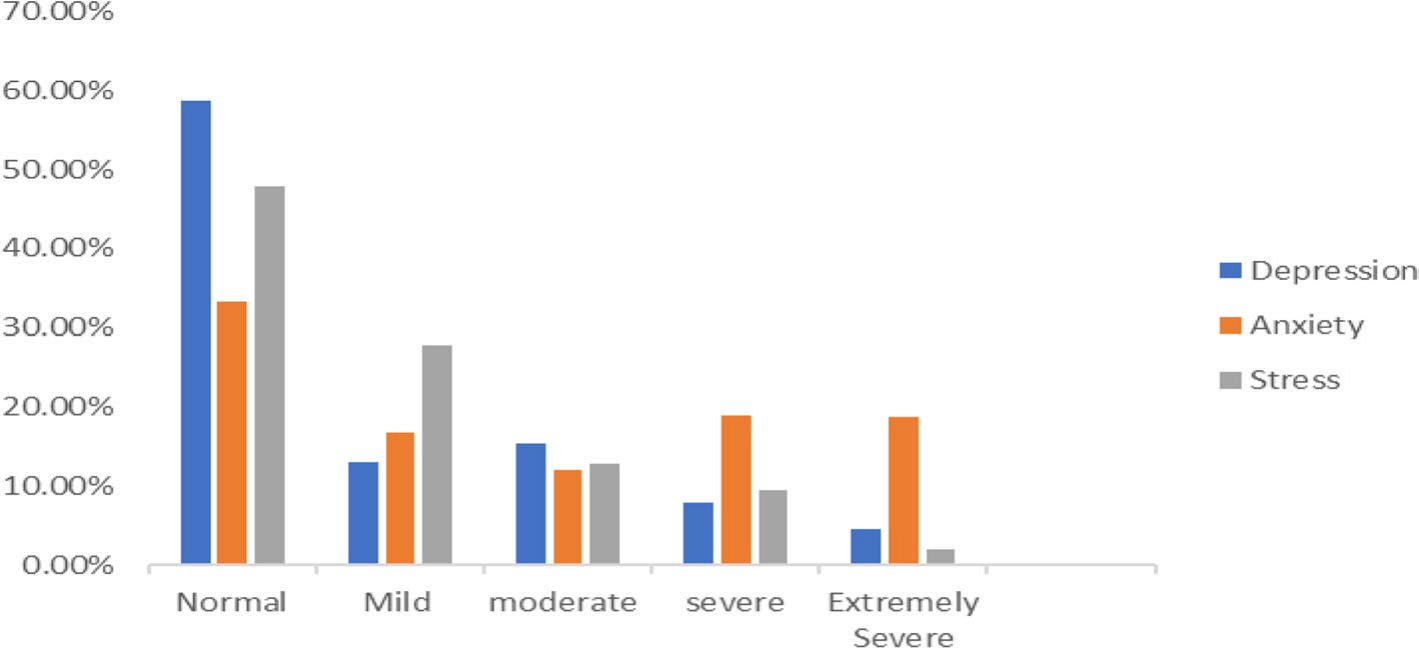
Severity of depression, anxiety, and stress for participants among high school students in Northwest Ethiopia, 2021 (*n*=849)

The overall prevalence of depression, anxiety, and stress in this study was found to be 41.4% (95%, CI: 38.0, 44.8%), 66.7% (95%, CI: 66.4, 66.9%), and 52.2% (95%, CI: 49.1, 56.0%), respectively. One hundred-nine (13.5%) respondents had mild depression, 124 (15.3%) had moderate depression, 65 (8.0%) had severe depression, and 37 (4.6%) had extremely severe depression. Similarly, 117 (14.4%) respondents had mild stress, 197 (24.3%) had moderate stress, 98 (12.1%) had severe stress, and 14 (1.7%) had extremely severe stress (Fig. 1).

### Associated factors of depression

In this study, sex, age, father’s educational status, grade, absenteeism, comorbid chronic medical illness, family history of mental illness, social phobia, and chewing Khat were factors associated with depression at a *p*-value less than 0.2 in bivariant analysis. Finally, multivariate analysis revealed that sex, khat chewing, and social phobia were found to be significantly associated with depression with 95% of CI and at a *p*-value less than or equal to 0.05. Female students were 1.3 times more likely to develop depression as compared with male students (AOR = 1.304, 95% CI = 1.006–1.849) and higher risk khat chewers were 5.6 (AOR = 5.595, 95% CI = 2.357–11.132) times more likely to have depression than those who were lower risky chewers. Another associated factor with depression was having social phobia (AOR = 1.416, 95%CI = 1.045–1.919), which is 1.4 times more likely to have depression than those who did not (Table 4).

**Table 4:** Bi-variable and multi-variable regression analysis showing associations between independent variables and depression among high school students in Northwest Ethiopia, 2021 (*n* = 810)

Variables	Category	Depression		COR and 95% CI	AOR and 95% CI	*p*-value
		Yes	No			
Sex	**Female**	**198**	**229**	**1.553(1.170–2.059)**	**1.304(1.006–1.849)**	0.046*
	Male	137	246	1	1	1
Age	≤19	233	304	1.285(0.953–1.732)	1.149(0.824–1.601)	0.413
	≥20	102	171	1	1	1
Grade	9	120	135	1.264(0.844–1.892)	1.123(0.729–1.276)	0.600
	10	75	129	0.827(0.539–1.268)	0.763(0.471–1.218)	0.257
	11	76	120	0.901(0.586–1.384)	0.822(0.522–1.296)	0.399
	12	64	91	1	1	1
Father educational status	unable to read and write	104	111	1.642(1.008–5.202)	2.251(0.961–5.170)	0.061
	can read and write but not attending formal education	184	274	1.642(0.522–3.221)	1.694(0.731–3.925)	0.219
	learned 1–8 grade	26	49	1.544(0.535–4.456)	1.493(0.576–3.872)	0.409
	secondary education	12	19	1.544(0.535–4.456)	1.699(0.508–5.084)	0.343
	Diploma and above	9	22	1	1	1
Absenteeism	Yes	48	51	1.390(0.912–2.120)	1.149(0.805–1.967)	0.314
	No	287	424	1	1	1
Medical illness	Yes	29	29	1.458(0.854–2.488)	1.277(0.718–2.272)	0.405
	No	306	446	1	1	1
Family history of mental illness	Yes	44	47	1.377(0.889–2.152)	1.134(0.709–1.813)	0.601
	No	291	428	1	1	1
Social phobia	**Yes**	**146**	**156**	**1.580(1.184–2.108)**	**1.416(1.045–1.919)**	0.025*
	No	189	319	1	1	1
Chewing khat	**Higher risk**	**31**	**7**	**6.955(3.023–9.324)**	**5.595(2.357–11.132)**	0.000***
	Moderate risk	13	11	1.856(0.820–4.198)	1.944(0.846–4.469)	0.117
	Low risk	291	457	1	1	1

*p-value (0.01–0.05) **p-value (0.01–0.001) and ***p value ≤0.001* degree of freedom =8; Hosmer-Lemeshow test = 0.78

### Associated factors of anxiety

On bi-variable logistic regression analysis, anxiety was found to be associated with students’ original residency, living conditions, mother’s educational status, grade, suicidal ideation, academic performance, family history of mental illness, alcohol drinking, and cigarette smoking. On multivariate analysis, Comorbid chronic medical illness, family history of mental illness, and smoking cigarette were significantly associated with anxiety. Participants who had a history of known chronic medical illness were about 2 (AOR = 2.099, 95%CI = 1.045–4.218) times more likely to develop anxiety when compared with those who had no medical illness and had a family history of mental illness (AOR = 1.777, 95%CI = 1.028–3.2073), which is 1.8 times more likely to have anxiety than those who had not. Higher-risky cigarette smokers were 4.8 times more likely to have anxiety than those who were lower-risky smokers (AOR = 4.777, 95% CI = 1.407–7304) (Table 5).

**Table 5:** Bi-variable and multi-variable regression analysis showing associations between independent variables and anxiety among high school students in Northwest Ethiopia, 2021 (*n* = 810)

Variables	Categories	Anxiety		COR and 95% CI	AOR and 95% CI	*p*-value
		Yes	No			
Residency	Rural	388	183	1.214(0.884–1.666)	1.211(0.860–1.760)	0.274
	Town	152	87	1	1	1
Living conditions	Relatives	37	12	1.650(0.843–3.230)	1.739(0.982–2.792)	0.072
	Alone	38	15	1.356(0.730–2.520)	1.441(0.760–2.752)	0.263
	With single parent	54	23	1.257(0.751–2.103)	1.241(0.727–2.116)	0.428
	With two parent	411	220	1	1	1
Grade	9	186	69	1.483(0.965–2.278)	1.274(0.810–2.004)	0.295
	10	121	83	0.802(0.521–1.235)	0.764(0.486–1.202)	0.244
	11	133	63	1.161(0.744–1.812)	1.104(0,695–1.755)	0.676
	12	100	55	1	1	1
Mother educational status	unable to read and write	393	181	0.543(0.151–1.947)	0.786(0.204–3.026)	0.726
	can read and write but not attending formal education	106	63	0.421(0.114–1.548)	0.607(0.153–2.401)	0.477
	learned 1–8 grade	21	15	0.350(0.084–1.460)	0.607(0.134–2.749)	0.517
	secondary education	8	8	0.250(0.050–1.239)	0.331(0.062–1.774)	0.197
		12	3	1	1	1
Academic performance	Poor	46	22	1.444(0.7212.891)	1.366(0.654–2.855)	0.407
	Sufficient	349	155	1.555(0.934–2.588)	1.610(0.937–2.767)	0.085
	Good	Diploma and above 103	64	1.111(0.630–1.959)	1.177(646–2.142)	0.595
	Very good	42	29	1	1	1
**Comorbid chronic medical illness**	**Yes**	**47**	**11**	**1.245(1.145–4.402)**	**2.099(1.045–4.218)**	0.037*
	No	493	259	1	1	1
Current suicidal ideation	Yes	33	10	1.692(0.821–3.488)	1.394(0.645–3.014)	0.398
	No	507	260	1	1	1
**Family histry of mental ilness**	**Yes**	**71**	**20**	**1.892(1.126–3.181)**	**1.777(1.028–3.073)**	0.04*
	No	469	250	1	1	1
Alcohol drinking	Higher risk	47	17	1.502(0.842–2.678)	1.236(0.664–2.301)	0.503
	Moderate risk	88	33	1.449(0.940–2.232)	1.328(0.845–2.089)	0.219
	Low risk	405	220	1	1	1
**Cigarette smoking**	**Higher risk**	**30**	**3**	**5.324(1.610–9.6730**	**4.777(1.407–7304)**	0.012*
	Moderate risk	10	4	2.130(0.705–5.435)	2.026(0.664–2.301)	0223
	Low risk	494	263	1	1	1

*p-value (0.01–0.05) **p-value (0.01–0.001) and ***p value ≤0.001; degree of freedom 9; Hosmer-Lemeshow test = 0.81

### Associated factors of stress

On bi-variable analysis, stress was found to be associated with age, grade, residency, marital status, academic performance, mother’s educational status, drinking alcohol, chewing khat, and social support. However, drinking alcohol, social support, and residency of respondents were significantly associated with stress in the final model. Students who come from rural areas were 1.4 times more likely to develop stress than those from urban areas (AOR = 1.395, 95%CI: 1.010–1.925). The odds of having stress were 1.8 times more prevalent in highly risky current alcohol drinkers as compared to low-risk alcohol drinkers (AOR 1.828, 95%CI = 1.012–3.303), and students who had poor social support were about 1.7 times more likely to develop stress when compared to those who had strong social support (AOR 1.7391, 95% CI = 1.203–2.515) (Table 6).

**Table 6:** Bi-variable and multi-variable regression analysis showing associations between independent variables and stress among high school students in Northwest Ethiopia, 2021 (*n* = 810)

Variables	Categories	Stress		COR and 95% CI	AOR and 95% CI	***p***-value
		Yes	No			
Age	≤19	292	245	1.236(0.923–1.655)	1.122(0.813–1.547)	0.485
	≥20	134	139	1	1	1
Grade	9	140	115	1.403(0.940–2.095)	1.073(0.695–1.656)	0.751
	10	110	94	1.349(0.887–2.051)	1.130(0.717–1.7820	0.598
	11	104	92	1.303(0.854–1.988)	1.122(0.714–1.763)	0.618
	12	72	83	1	1	1
**Residency**	**Rural**	**318**	**253**	**1.525(1.125–2.065)**	**1.395(1.010–1.925)**	0.044*
	Town	108	131	1	1	1
Marital status	Divorced	8	2	3.636(0.689–9.194)	1.128(0.932–2.345	0.092
	Single	396	362	0.994(0.534–1.853)	1.120(0.574–2.186	0.739
	Married	22	20	1	1	1
Mother educational status	unable to read and write	298	276	0.393(0.124–1.247)	0.340(0.098–1.185)	0.090
	can read and write but not attending formal education	90	79	0.414(0.127–1.353)	0.384(0.108–1.377)	0.142
	learned 1–8 grade	18	18	0.364(0.097–1.358)	0.364(0.088–1.500)	0.162
	secondary education	7	9	0.468(0.103–2.120)	0.569(0.116–2.799)	0.488
	Diploma and above	4	11	1	1	1
Academic performance	Poor	44	24	1.886(0.954–3.726)	1.645(0.803–3.371)	0.174
	Sufficient	277	227	1.255(0.763–2.064)	1.221(0.726–2.506)	0.452
	Good	70	97	0.742(0.425–1.296)	0.714(0.399–1.279)	0.257
	Very good	35	36	1	1	1
**Alcohol**	**High risk**	**44**	**20**	**2.179(1.251–3.782)**	**1.828(1.012–3.303)**	0.045*
	Moderate risk	68	53	1.271(0.859–1.880)	1.054(0.698–1.592)	0.802
	Low risk	314	311	1	1	1
Khat	High risk	22	16	1.276(0.660–2.468)	1.064(0.532–2.128)	0.861
	Moderate risk	16	8	1.856(0.785–4.388)	1.541(0.598–3.372)	0.370
	Low risk	388	360	1	1	1
Cigarette	High risk	22	11	1.852(0.886–3.874)	1.491(0.675–3.291	0.323
	Moderate risk	11	9	1.132(0.464–2.763)	1.057(0.405–2.759)	0.911
	Low risk	393	364	1	1	1
**Social support**	**poor**	**143**	**106**	**1.757(1.233–2.504)**	**1.739(1.203–2.515)**	0.003**
	Moderate	174	136	1.667(1.192–2.331)	1.126(0.981–1.451)	0.081
	Strong	109	142	1	1	1

* *P* value (0.01–0.05), **p value (0.01–0.001) and ***p value ≤0.001; degree of freedom 9; Hosmer-Lemeshow test = 0.75

## Discussion

The presence of depression, anxiety, and stress harms academic performance in the school by interfering with their emotional, cognitive, and social abilities, and increasing school absentees. This leads to a significant impairment of the emotional, psychological, social, and physical well-being of students. In this study, the prevalence of depression, anxiety, and stress and their possible association with different factors were assessed. The result revealed that a remarkable proportion of students had depression, anxiety, and stress symptoms.

The findings of the current study showed that the prevalence of depression among high school students was found to be 41.4% (95% CI: 38.0, 45.1%), which was consistent with the findings of other studies done in two different studies of Saudi Arabia, Malaysia, and China reported to be 38.2, 41.5 39.7, and 43.3% respectively [2, 15, 17, 20]. However, the prevalence of depression in this study was higher than previous research findings done among high school adolescents in Manipur India, and Iraq, which was 19.5 and 29.4%, respectively [1, 16]. The possible reason for the variation may be due to a difference in the number of female participants between the current and previous studies. More than half of the participants in this study are females, and of these, 46.4% have depression. On the other hand, the current study finding is lower than the previous study done in Chandigarh India (65.53%) [18]. The possible reason for this discrepancy might be the difference in the study population.

The overall prevalence of anxiety in this study was found to be 66.7% (CI: 63.7, 70.2%), which is almost similar to a study conducted on Saudi Arabian female students and Malaysian high school students, which was 66.2 and 67.1%, respectively [2, 15]. However, the prevalence of anxiety in this study was higher than that reported in Saudi Arabian school boys, a study conducted in 2014 among Indian adolescents, and Iraqi high school students reported to be 48.9, 24.4, and 40.6% respectively [1, 16, 20]. The variation might be due to differences in sociocultural, socioeconomic, type of study population, and availability of health facilities between those countries and Ethiopia. People living in low socioeconomic countries like Ethiopia could have poor health care infrastructure and a shortage of trained health staff that delivers inadequate health care services. In turn, anxiety might not be early identified and treated [27, 28]. On the other hand, the prevalence of anxiety in this study is lower than in a study conducted in 2017 among school-going adolescents in India, where it was found to be 80.85% [18].

The prevalence of stress in this study was found to be 52.6% (95%, CI: 49.1, 56.0%), which agrees with a previous study conducted on Saudi Arabia females students and Iraq high school adolescents, which were 52.5 and 51.1%, respectively [2, 16]. However, the prevalence of stress in this study was higher than in the studies conducted on Saudi Arabian school boys, in two different areas of India and Malaysia high school students reported to be 35.5, 21.1, 47.02, and 44.9% respectively [1, 15, 18, 20]. The variation could be a difference in the participants’ social support systems. In the current study, about 45% of students have poor social support. Therefore, poor social support has been associated with physiological and neuroendocrine indices of heightened stress reactivity [29].

Regarding factors affecting depression, female students were 1.3 times more likely to develop depression as compared with male students. This finding is supported by other studies done in Ghana, Chandigarh India, and Malaysia among high school students [15, 18, 30]. The possible reasons could be that women more often present with internalizing symptoms and more sensitivity to interpersonal relationships, and specific forms of depression-related illness, including premenstrual dysphoric disorder, and postpartum depression, that are associated with changes in ovarian hormones and could contribute to the increase in women [31, 32].

Another associated factor with depression was having a social phobia, which is 1.4 times more likely to have depression than those who did not. Similar findings were reported in a comparative study of the general population of the United Kingdom, Germany, Italy, Spain, and Portugal [33]. The reason could be that socially anxious students may have trouble making friends and maintaining close relationships, which can even result in missed opportunities. This often leads to frustration, feelings of hopelessness, and isolation, which resembles depression [34].

Higher-risky khat chewers were about 5.6 times more likely to have depression than lower-risky chewers, which agrees with a previous study conducted on Jimma University staff and Tepi Town residents in Ethiopia [35, 36]. The possible reason is that the primary psychoactive ingredients of khat cathinone and cathine, stimulate the release of cortisol, norepinephrine, and dopamine. Consequently, the respondents initially experience stimulator effects such as excitement and talkativeness. Then, they develop excessive worry, depression, and tension [37]. The other possible justification is the socioeconomic problems due to the increased demand for money to buy khat [38].

Higher-risk cigarette smokers were 4.8 times more likely to have anxiety than those who were lower-risk smokers. This is supported by a previous study conducted among Medical Undergraduate Students of Arsi University, Tepi Town Residents, and Mettu Karl Referral Hospital [21, 36, 39]. The financial burden of affording it, concern about the increased severity of smoking, and a sense of stigma from classmates and people influenced to quit smoking are all possible reasons for an individual becoming more anxious [40].

Students with a positive family history of mental illness had about 1.8 times more odds of having anxiety as compared with students who had no family history of mental illness. This is supported by studies being done in Pakistan [41]. Having a mental illness in the family may adversely affect the quality of caregiving, family interactions, and, the use of negative parenting styles, such as less affection, supervision, and autonomy. These consequences of family stress may in turn contribute to maladaptive perception and appraisal of life events and ways of coping with stressful situations, and eventually lead to anxious behavior [42, 43].

The present study also showed that anxiety was significantly associated with the presence of a known chronic medical illness among high school students. The odds of having anxiety were two times more common among students having a history of known chronic medical illness as compared with their counterparts. This was supported by two previous studies done among university students in Egypt and Sri Lanka [44, 45]. Because of the nature of chronic illnesses, whether it be increased hospitalization, excessive worry, or hormonal changes, having a chronic illness increases the likelihood of developing a mental illness, mainly anxiety disorders. Students with chronic diseases may never get better, and their illnesses may never go away, disrupting their lives in several ways. Pain and fatigue may become a regular part of their day, as may fears about hospitalization, diagnosis, painful procedures, and a shortened lifespan, all of which make students more anxious [46].

Compared to low-risk alcoholic drinkers, the odds of stress were nearly two times more common among highly risky alcoholic drinkers. This was supported by a previous study conducted among medical undergraduate students at Arsi University [21]. The possible reason could be that, as we know, drinking alcohol can cause some general life stress, such as causing relationship problems, legal problems, and trouble with family and work. All this may negatively affect thoughts, feelings, and actions, which contributes to the development of stress over time among students [47].

Participants who had poor social support were 1.7 times more stressed than those who had strong social support. This was supported by a previous study conducted among high school students in Ghana [30]. This is because Adolescents with poor social support can’t get opportunities like advice, guidance, encouragement, acceptance, emotional comfort, and tangible assistance such as financial help. Thus, students can’t be comforted when they are faced with a wide range of life stressors, and they can be extremely stressed in their efforts to manage these challenges [29].

Finally, stress was nearly two-thirds more prevalent among rural students than among students found in town. This was supported by a study conducted among Arsi University undergraduates [21]. Since there is no high school in the countryside of Ethiopia, students from rural areas attend high school education in a new environment, moving to nearby towns. Therefore, making a major move can be a stressful time for many students. A new environment, new classes, new teachers, and new routines can all be stressful for rural students, and take time to adjust to their lives. The individual continually recognizes and evaluates stimuli in a stressful environment, and undergoes physical and psychological changes to adapt to the needs of the environment.

## Strengths and limitations of the study

The fact that this study was conducted at six high schools (multisite) with a large sample size makes it more generalizable than any other study, which is its strength. However, this study also had some limitations that need to be taken into consideration. Self-administered questionnaires were used to collect the data, therefore reporting bias may exist. Additionally, some questions assessed history, which is subject to recall bias. Since the study was cross-sectional, a cause-and-effect relationship was challenging to establish.

## Conclusion and recommendation

Overall, the prevalence of depression, anxiety, and stress was higher among high school students. Female sex, chewing khat, and social phobia were associated with depression. On the other hand, smoking cigarettes, having chronic medical illness and a family history of mental illness associated with anxiety, being a highly risky alcoholic drinker, having poor social support, and having rural residency are positively associated with stress. Hence, the Ethiopian Ministry of Health is better to do in collaborate with the Ministry of Education for extending mental health services to all high schools, and strengthening the existing counseling services are recommended so that early detection of depression, anxiety, and stress can be done. For researchers who are interested to study in this area, it is better to conduct longitudinal research to identify the cause-and-effect relationship between depression, anxiety, and stress with different factors.

## Data Availability

Data is available upon request from the corresponding author.

## Abbreviations

AOR: Adjusted Odds Ratio
ASSIST: Alcohol, Smoking, and Substance Involvement Screening Test
CI: Confidence Interval
COR: Crude Odds Ratio
KM: Kilo Meter
MI: Mental Illness
OR: Odds Ratio
SPIN: Social Phobia Inventory
USA: United States of America
WHO: World Health Organization
